# Seroprevalence of bovine herpesvirus-1 antibodies in bovines in five districts of Uttarakhand

**DOI:** 10.14202/vetworld.2017.140-143

**Published:** 2017-02-04

**Authors:** Vipul Thakur, Mahesh Kumar, R. L. Rathish

**Affiliations:** Department of Epidemiology and Preventive Medicine, College of Veterinary and Animal Science, G.B. Pant University of Agriculture and Technology, Pantnagar, Udham Singh Nagar - 263 145, Uttarakhand, India

**Keywords:** avidin biotin enzyme-linked immunosorbent assay, bovine herpesvirus-1, seroprevalence

## Abstract

**Aim:**

This study was conducted to know the status of bovine herpesvirus-1 (BHV-1) antibodies in the bovines of the selected area of Uttarakhand.

**Materials and Methods:**

A total of 489 serum samples, 392 of cattle and 97 of buffaloes were randomly collected from the unvaccinated bovine population of five districts viz., Dehradun, Haridwar, Nainital, Pithoragarh, and Udham Singh Nagar and were tested by avidin biotin enzyme-linked immunosorbent assay for BHV-1 antibodies.

**Results:**

The overall prevalence was observed to be 29.03%. At district level, the highest prevalence was recorded in Pithoragarh district (40.00%) while it was lowest in district Udham Singh Nagar (16.00%). The prevalence of BHV-1 antibodies was found to be higher in unorganized dairy units (31.02%) compared to organized farms (26.51%) in Uttarakhand. Buffaloes were found to have greater prevalence (38.14%) than cattle (26.78%) while on sex-wise basis; it was found that more females (30.08%) were harboring antibodies to the virus than males (16.21%).

**Conclusion:**

The study revealed that the population in the area under study has been exposed to BHV-1 and hence prevention and control strategies must be implemented.

## Introduction

Bovine herpesvirus 1 (BHV-1), which infects domestic and range cattle, is associated with several clinical conditions including infectious bovine rhinotracheitis (IBR), infectious pustular vulvovaginitis, balanoposthitis, conjunctivitis, and generalized disease in newborn calves. BHV-1 has been classified into subtypes 1.1, 1.2a, and 1.2b using restriction enzyme analysis. Subtype 1.1 is associated with respiratory disease while subtypes 1.2a and 1.2b are implicated in infectious pustular vulvovaginitis/infectious balanoposthitis syndrome and cause mild respiratory disease [1].

The virus is transmitted primarily through aerosol or genital contact. Clinical signs are generally mild, and the virus does not cause high mortality, infection results in a latent state and lifelong infection. Reactivation of latent BHV-1 infections can occur due to corticosteroid treatment or stress due to transportation, overcrowding or adverse weather conditions. The productivity and reproductivity of the animals is greatly decreased as an outcome of the disease [1-3].

Various workers have published reports regarding the prevalence of this disease from different parts of India [4-7].

Enzyme-linked immunosorbent assay (ELISA) is a sensitive and specific test in terms of detection of the low level of antibody for several viral diseases; this has been extensively used in recent last by many workers to monitor the seroprevalence of IBR in cattle population [8]. There are many reports regarding the use of various types of ELISA to detect BHV-1 antibody in serum samples of cattle, and these tests can also be used to detect acute, convalescent, and latent stages of disease [9].

This investigation was carried out to know the prevalence of BHV-1 antibodies in bovines of Uttarakhand using avidin biotin ELISA (AB-ELISA) and to determine the significance of risk factors such as management, species, and sex associated with the BHV-1 prevalence.

## Materials and Methods

### Ethical approval

As per CPCSEA guidelines, a study involving clinical samples does not require the approval of Institute Animal Ethics Committee.

### Sample collection

A total of 489 serum samples were collected from cattle and buffaloes of five districts of Uttarakhand, *viz*., Dehradun, Haridwar, Nainital, Pithoragarh, and Udham Singh Nagar (Table-1). Total 216 serum samples were collected from organized herd, of which 208 were of cattle, (178 of cows and 30 of breeding bulls) and eight from buffalo breeding bulls. From unorganized sector, total 274 serum samples were collected out of which 185 were of cattle and 89 from buffaloes. Serum samples were collected as per standard procedure. Serum samples were stored at −20°C until assay procedure.

**Table 1:** Details of sample collected and seroprevalence.

Categorization of datas	Cattle		Buffalo		Total	
		
	ST	SP (%)	ST	SP (%)	ST	SP (%)
District						
Udham singh nagar	30	5 (16.66)	20	3 (15.00)	50	8 (16.00)
Nainital	70	17 (24.28)	69	32 (46.37)	139	49 (35.25)
Dehradun	207	55 (26.57)	8	2 (25.00)	215	57 (26.51)
Haridwar	50	14 (28.00)	-	-	50	14 (28.00)
Pithoragarh	35	14 (40.00)	-	-	35	14 (40.00)
Total	392	105 (26.78)	97	37 (38.14)	489	142 (29.03)
Sex-wise						
Male	29	4 (13.79)	8	2 (25.00)	37	6 (16.21)
Female	363	101 (27.82)	89	35 (39.32)	452	136 (30.08)
Total	392	105 (26.78)	97	37 (38.14)	489	142 (29.03)
Management system						
Organized	207	55 (26.57)	8	2 (25.00)	215	57 (26.51)
Unorganized	185	50 (27.02)	89	35 (39.32)	274	85 (31.02)
Total	392	105 (26.78)	97	37 (38.14)	489	142 (29.03)

### ELISA

AB-ELISA kit to test 500 samples was procured from NIVEDI, Bengaluru, and test was carried out according to manufacturer’s instructions.

Briefly, all test serum samples and control serum samples were diluted to 1:100 in blocking buffer (1 g of bovine gelatin powder was dissolved in 100 ml of phosphate-buffered saline [PBS] ×1. To this solution 50 µl of Tween 20 was added. It was prepared fresh every time). BHV-1 antigen-coated plate was brought to room temperature. 100 µl of diluted control and test sera were added in duplicates to the wells. The plate was incubated on shaker for 1 h at 37°C. Thereafter was washed 3 times with washing buffer (50 µl of Tween 20 to 100 ml of PBS ×1). Then, 100 µl of biotin anti-immunoglobulin G conjugate diluted 1:30000 in blocking buffer was added to all the wells. Plate was again incubated on shaker for 1 h at 37°C and washed 3 times with washing buffer. 100 µl of Avidin-horseradish peroxidase conjugate diluted to 1:20,000 in blocking buffer was added to each well. Plate was again incubated on shaker for 20 min at 37°C and then washed 3 times with washing buffer. Then, 100 µl of chromogen/substrate was added to all the wells. The plate was then incubated at room temperature for 8-10 min. Then, 50 µl of stopping solution (1 N sulfuric acid) was added to all the wells. The plate was read in the ELISA plate reader at 492 nm. Results of test sera were expressed as percent positivity (PP) values calculated as shown below:

PP = (Average OD of sample/Average OD of strong serum positive) × 100

The test sera with PP values ≥45 were considered positive for IBR antibodies positive.

### Statistical analysis

Statistical analysis of data was performed as per the method described by Snedecor and Cochran [10].

## Results

Out of 489 serum samples screened, 142 (29.03%) were found to be positive AB-ELISA for BHV-1 antibodies (Table-1). At district level, seroprevalence of IBR antibodies was highest in Pithoragarh district (40.00%) and lowest in district Udham Singh Nagar (16.00%).

Seroprevalence was higher in unorganized dairy units (31.02%) compared to organized farms (26.51%) in Uttarakhand. At species level also higher prevalence level was observed in both cattle and buffalo from unorganized sector compared to those from organized sector. However, the dependency of prevalence on management system followed was nonsignificant at p≤0.05. Species wise seroprevalence was significantly higher in buffaloes (38.14%) than cattle (26.78%) at p≤0.05. Overall, sex-wise seroprevalence indicated that more females (30.08%) were affected by BHV-1 than males (16.21%). Dependency of prevalence on sex was nonsignificant at p≤0.05. At species level also for prevalence percentage in both cattle and buffaloes was more in females (27.82% and 39.32%, respectively) than males (13.79% and 25.00%, respectively).

## Discussion

In Uttarakhand, in earlier studies, Jain *et al*. [11] and Nandi *et al*. [12] have reported lower seroprevalences, 10.39% and 22.3%, respectively. In an independent study using complement ELISA (cELISA), Thakur *et al*. [13] have also reported lower prevalence 18.15% involving 200 samples while quite higher prevalence (40.71%) have been recorded by Kollannur *et al*. [14]. The difference in the prevalence rates may be attributed to difference in year of study, area/district selected for sample collection, variation in sample size, and test employed.

In agreement with this study, Thakur *et al*. [13] also reported the highest prevalence in Pithoragarh district using cELISA. However, the study was conducted on a lower sample size of 200 and covered only three districts, *viz*., Udham Singh Nagar, Dehradun, and Pithoragarh. Although the prevalence of different districts differed significantly, however, there is a need to study in detail the other factors such as migration of animal, breeding practices, geographical location, and climatic conditions and their effect on virus spread before associating the occurrence on antibodies in particular district. Much higher prevalence rates from different parts of India and world were reported by various workers [15-20]. Whereas, the lower seroprevalence rate was observed by Trangadia *et al*. [6], Das *et al*. [8], Singh and Sinha [21], and Singh *et al*. [22] from different parts of India with respect to this study.

Similar to our observation Rajesh *et al*. [23] in Kerala also reported higher prevalence in unorganized herds (18.75%) than organized herds (13.13%). Contrary to the present findings, Singh and Yadav [5] observed significantly lower prevalence in unorganized herds (13.2%) as compared to organized herds (43.3%) with an overall seropositivity of 32.31%. Singh *et al*. [22] also compared the seroprevalence between organized and unorganized herd and found that the former was having a higher prevalence of Likewise, Ganguly *et al*. [24] and Sathiyabama *et al*. [25] also reported higher prevalence in unorganized dairy herds than organized. The possible reason for higher prevalence in unorganized dairy herds might due to the practice of natural breeding with bulls whose disease status is not known. The natural breeding with bulls without knowing their disease status could be responsible for the rapid spread of the disease as also opined by Romero-Salas *et al*. [16]. Gonzalez-Garcia *et al*. [26] indicated a lack of specific cattle infrastructure and beef crossbreeding as important risk factors associated with BHV-1 infection in Spain along with herd size, history of reproductive disorders, purchase of replacements and proximity to an urban area.

Similar to our observations, on species wise prevalence, other researchers have reported greater prevalence in buffaloes than cattle. In Uttarakhand, Thakur *et al*. [13] have also reported lower prevalence in cattle (17.44%) than buffaloes (25.00%) using competitive ELISA. The observation of this study also concurred with the reports of Renukaradhya *et al*. [27] who also observed lower seropositivity in cattle (50.9%) than buffaloes (52.5%) in India. Similarly, the lower prevalence rate of BHV-1 antibodies in cattle with respect to buffaloes have been reported by Krishnamoorthy *et al*. [20], Nandi *et al*. [28], and Trangadia *et al*. [15] in India. In contrast to the present results, Dwivedi [29] and Jain *et al*. [11] in independent studies have reported higher prevalence in cattle (11.27% and 10.75%, respectively) compared to buffaloes (10.61% and 8.89%, respectively) in Uttarakhand. Jain *et al*. [30] in Gujarat; Sharma *et al*. [31] in Uttar Pradesh and Verma *et al*. [7] in Uttar Pradesh have also reported greater prevalence rate in cattle population than buffaloes.

Observations of this study in context to sex wise prevalence were in agreement to study of Thakur *et al*. [13] who have also observed higher prevalence rate in females (19.02%) than males (16.22%) in Uttarakhand. Similarly, in an earlier study, Jain *et al*. [11] in Uttarakhand have also reported the greater prevalence of BHV-1 antibodies in females (12.35%) than males (5.80%), and this was evident even at the species level for both cattle and buffaloes. Saravanajayam *et al*. [32] also observed the higher prevalence of IBR antibodies in female (67.92%) than in male (33.33%). Similarly, Nandi *et al*. [33]; Krishnamoorthy *et al*. [20] in southern India; Singh and Sinha [21] in Bihar and Sharma *et al*. [31] in Uttar Pradesh also reported greater percent prevalence in females than males. The possible reason for higher seropositivity in females might be due to the use of infected semen/seropositive bull for insemination/breeding [16]. Contrary to our observation, Verma *et al*. [7] had observed more males to be seropositive than females in a study in Uttar Pradesh.

## Conclusions

This study revealed that the BHV-1 is strengthening its roots in different parts of Uttarakhand. The present findings warrant that a suitable action plan should be implemented to control the spread of virus in the state.

## Authors’ Contributions

A collection of serum samples, screening of samples by ELISA was done by VT. The entire work was done under supervision of MK. Data were analyzed by VT. The manuscript was prepared by VT, and corrections/modifications were done by MK and RRL. All authors read and approved the manuscript.
